# Network analysis of loneliness, mental, and physical health in Czech adolescents

**DOI:** 10.1186/s13034-025-00884-7

**Published:** 2025-03-28

**Authors:** Zdenek Meier, Jakub Helvich, Jana Furstova, Lukas Novak, Dana Purova, Radka Zidkova, Peter Tavel

**Affiliations:** Olomouc University Social Health Institute, Palacky University in Olomouc, Univerzitni 244/22, 771 11 Olomouc, Czech Republic

**Keywords:** Loneliness, Adolescents, Mental health, Physical health, HBSC, Network analysis

## Abstract

**Background:**

The increasing urgency to address rising loneliness among adolescents has become a critical issue, underscoring the need for further studies on its association with mental and physical health. The objective was to examine the changes in loneliness and its relation to mental and physical health issues in three adolescent age groups.

**Methods:**

A total sample of 14,588 Czech pupils (50.7% boys, mean age 13.6 ± 1.7 years) in grades 5, 7 and 9 was used from a representative dataset of the Health Behaviour in School-aged Children (HBSC) study. The network analysis based on undirected graphical models was used as an exploratory technique to assess and test the structure of the data.

**Results:**

The association between loneliness and health decreased with age. There was a significant positive association between loneliness, feeling low, and irritability. No significant direct association between loneliness and physical health complaints was found.

**Conclusion:**

Further studies, preferably of longitudinal character, are needed to confirm the changes in associations between loneliness and mental and physical health outcomes.

**Supplementary Information:**

The online version contains supplementary material available at 10.1186/s13034-025-00884-7.

## Introduction

Loneliness, extensively explored in developmental and evolutionary contexts, is defined as the distressing gap between desired and actual social connections [1, 2]. It differs from aloneness—often referred to as solitude—which can be neutral or positive when voluntarily chosen and may foster self-reflection and personal growth [3, 4]. Children’s ability to distinguish loneliness from solitude evolves with age: younger children often conflate being alone with loneliness, whereas older children and adolescents gradually recognize the motivational differences that set voluntary solitude apart from involuntary isolation [4]. This distinction highlights a complex interplay of emotional, social, and evolutionary factors [5, 6]. While loneliness stems from unmet social needs [7, 8], solitude can be a conscious, beneficial choice [9, 10]. Recognizing these nuances is critical for addressing recent spikes of loneliness in the young populations.

The rising prevalence of loneliness has become a critical issue [7, 11], garnering attention from researchers due to its pervasive and far-reaching impacts. Specifically alarming is a global increase in loneliness among adolescents [12–15], a youth population undergoing crucial developmental changes while facing a variety of unique social challenges [16, 17]. The COVID-19 pandemic has significantly exacerbated the rising concern of loneliness, as social distancing measures and lockdowns have significantly disrupted interpersonal interactions, leaving many adolescents isolated from their peers [18, 19]. The long-term impacts of the pandemic abetted by disrupted social development have led to a continuous increase in loneliness among adolescents [18, 20, 21].

Loneliness is a widespread phenomenon, with more than 80% of individuals under the age of 18 and 40% of those over 65 reporting experiences of loneliness [7]. The Czech Republic, along with the Netherlands, Croatia, and Austria, reported lowest levels of loneliness according to a 2022 EU-wide survey, with prevalence rates around 10% [22]. However, a global increase in the prevalence of loneliness among adolescents has been reported in various studies even before the pandemic [12–14]. In the United States, surveys conducted by the health insurance provider CIGNA reported prevalence rates ranging from 38 to 48% in 2018, which rose by 1.7% points in 2019, with the steepest increases among younger generations [23, 24]. In the European Union loneliness grew from 12% in 2016 to 25% during the pandemic [25]. The Czech Republic has been one of the countries with the largest increases in reported school loneliness during the past two decades [15]. In the European Catholic region a country cluster with common cultural characteristics, the prevalence of high loneliness increased on average by 49.62% between the years 2000 and 2018, while in the Czech Republic, the prevalence increased by 87.41% compared other regional countries such as Poland (20.83%), Austria (42.17%), or France (72.37%) [15]. When assessing the changes between the years 2012 and 2018, the prevalence of high loneliness in the Czech Republic increased by 104.15%, compared to the region average of 76.55%. Extensive research has been conducted on loneliness in the Czech elderly population [26–30]. However, more recent studies on loneliness in the Czech adolescent population, particularly those using representative samples, remain relatively limited. This gap is worrying since loneliness can have far-reaching consequences on adolescents’ mental and physical health.

Previous research has highlighted some of the significant negative outcomes of loneliness on mental and physical health. Concerning mental health, studies have shown that loneliness is associated with depression, anxiety disorders, and increased stress levels [18, 31–34]. Additionally, previous research showed that loneliness is also related to low self-esteem, negative self-perceptions, and a higher propensity to self-harm, especially among younger populations [32, 35]. On the other hand, studies have also demonstrated significant positive links between loneliness and physical health issues such as stroke and coronary heart disease [36, 37], elevated blood pressure, weakened immune system, and increased inflammation [16, 38–40]. Further, loneliness can be related to various health risk behaviours [41]. Altogether, it can be concluded that loneliness is a key social determinant of general health [42, 43]. Nevertheless, recent evidence may cast doubt on a direct causal association between loneliness and health.

Many findings originate from observational or cross-sectional designs, complicating determinations of causality [36, 37, 44–47]. Confounding factors—such as socioeconomic status, existing health conditions, and the quality of social connections—further blur the direction of effect [45, 47, 48]. Some evidence indicates loneliness may simply mark poorer health, rather than cause it [49], whereas other studies suggest possible biological mechanisms (e.g., increased stress responses and inflammation) that could mediate the causal relation [50–52]. More longitudinal and cohort research is needed to clarify these relationships [33, 37, 53]. Nevertheless, two major research gaps remain, which previous studies have not fully resolved.

First, a crucial gap remains in understanding how the relationship between loneliness and health varies in different age groups of adolescents. Most studies have limited their scope to adolescents of a certain age, overlooking the physical, emotional, and social changes that occur during adolescence, which may influence the association between loneliness and health [54, 55]. Regarding mental health, younger adolescents might be more affected by peer rejection and social exclusion, while older adolescents may experience loneliness related to identity formation [1, 56]. Additionally, younger adolescents might exhibit immediate behavioural changes, such as disrupted sleep, whereas older adolescents are more prone to long-term health issues like chronic illnesses [57]. Therefore, examining multiple age groups can provide a better understanding of the negative outcomes of loneliness on adolescents’ overall health, leading to more targeted interventions [58].

Second, previous studies predominantly used statistical methods that could not capture the complex interplay between loneliness, and health. Traditional widely used approaches, such as univariate and multivariate regression analyses, may fail to reveal the complex bidirectional dynamics in the data [59, 60]. To overcome this shortcoming, psychological network analysis can be utilised. This approach to assessing complex psychological systems has emerged relatively recently [61]. In general, network analysis is a set of techniques investigating structure and patterns in the data. The analyses are based on several mathematical concepts, including graph theory and network optimisation. Using network analysis, researchers can identify key nodes and pathways, providing crucial data for developing effective intervention strategies to address loneliness and its relation to health [62].

Therefore, the objective of this study was to use exploratory network analysis to examine the changes in loneliness and its connection to mental and physical health in three age groups of the Czech adolescent population using a representative dataset from the Health Behaviour in School-aged Children (HBSC) study.

## Methods

### Participants and procedure

The data were collected from a nationally representative sample of Czech boys and girls as part of the 2022 HBSC study. This cross-sectional study, conducted in collaboration with the WHO, focuses on adolescents’ health, health-related behaviours, and socioeconomic determinants. The HBSC study has been conducted every four years since 1983/84 and currently includes 51 countries. The Czech Republic joined the study in 1993/94, making the 2022 data collection the eighth consecutive cycle. The data collection took place between May and June 2022. Between May and June 2022, regular in-class school attendance was restored in Czech schools without any major restrictions associated with COVID-19, and pupils were able to participate in classes without requiring special measures such as wearing masks or testing. According to the HBSC study protocol, schools were randomly selected after stratification by region, school size, and type (primary and secondary). Of the 272 Czech schools contacted, 246 agreed to participate, resulting in an 86.1% response rate. Classes from the 5th, 7th, and 9th grades, generally corresponding to ages 11, 13, and 15, were randomly selected, one per grade per school. Data were obtained from 14 879 pupils, with a response rate of 83.1%. The non-response was mainly due to illness (*n* = 1928) or other reasons such as sports or academic competitions (*n* = 1024), and 77 children declined to participate. During the data exclusion process, only questionnaires from pupils who matched their age to the year of school attended were selected for analysis. To ensure consistency of results, those who started school with a deferment or repeated a year, or, on the contrary, started school before the age of 6 were included in the final sample. A total of 168 respondents were removed from the sample due to age outside the permitted range. Additionally, a total of 111 questionnaires were excluded based on internal consistency (e.g., mutually exclusive answers, nonsensical answers to open-ended questions). The other 12 participants were excluded due to a large number of missing responses. The final Czech HBSC sample comprised 14 588 participants (50.7% boys, mean age 13.6 ± 1.7 years).

The data collection was conducted online using CAWI (Computer-Assisted Web Interviewing) via the unipark/TIVIAN platform. The collection took place in schools and was supervised by trained research assistants (*n* = 86) in the absence of teachers to minimise response bias. Respondents had one school lesson (45 min) to complete the survey. Participation in the survey was anonymous and completely voluntary. The study design was approved by the Ethics Committee of the Faculty of Physical Culture, Palacky University Olomouc (No. 14/2019), and conducted following the ethical requirements outlined in the Convention on Human Rights and Biomedicine (40/2000 Coll.).

### Measures

*Loneliness* was assessed as a self-report measure of general loneliness using the question: “During the past 12 months, how often have you felt lonely?”. The single-item measure has been used to assess the prevalence of loneliness in youth populations [63] and can be considered a reasonable proxy for loneliness degree. Past research suggested that a single-item loneliness measure similar to ours had an acceptable convergent and construct validity and reasonable correlation with a longer loneliness measure [64]. Respondents rate the item on a 5-point scale ranging from “never” (1) to “always” (5) with higher scores indicating greater perceived loneliness. The response options “never”, “rarely” and “sometimes” are considered normative, whereas “most of the time” and “always” indicate harmful levels that are associated with negative health outcomes [65].

*Self Rated Health (SRH)* was measured using a single-item self-report question: “Would you say your health is…?”. Respondents rate themselves on a 4-point scale from “excellent” (1) to “poor” (4). For this study, the item scoring was reversed, so that higher number means better health. This question, adapted from Kaplan & Camacho [66] measure of perceived health, was designed to assess the individual’s overall perception of their health.

*The Multiple Health Complaints Measure* is an eight-item scale which includes various self-report health symptoms, frequently occurring together [67] and provides an important indicator of mental health and well-being [68]. The measure consists of eight complaints: four somatic (headache, stomach ache, backache, feeling dizzy), and four psychological (feeling low, irritability or bad mood, feeling nervous, and difficulties falling asleep). Respondents answer the question: “In the last 6 months: how often have you had the following…?” with response options ranging from “About every day” (1) to “Rarely or never” (5). For this study, the item scoring was reversed, so that a higher number means more frequent occurrence.

Socioeconomic status was assessed with *the Family Affluence Scale (FAS)* [69]. The FAS is based on ownership of a car, a dishwasher, the number of computers and bathrooms in the household, the child having their own bedroom, and the frequency of holidays abroad. A summary score is categorised into three levels corresponding to the lowest 20%, the middle 60%, and the highest 20%.

### Data analysis

First, descriptive statistics (counts, proportions) were computed. Differences between groups were tested with the χ^2^ test. Next, the network analysis approach [61] was used as an exploratory technique to assess the structure of the data. The network analysis follows the methodology of graph theory, with nodes and edges representing variables and pairwise associations between them, while conditioning on all other variables in the dataset. In these undirected graphical models (also known as pairwise Markov random fields), unconnected nodes are conditionally independent. For the network estimation, the *qgraph* package in R [70] was employed, using polychoric correlations on ordinal data and the EBICglasso estimator [71], implementing regularization to eliminate spurious edges. Centrality parameters were explored to assess the position of nodes in the network. The stability and replicability of the network edges and centrality parameters were computed using the *bootnet* package in R [72]. A non-parametric bootstrap using 2500 samples with replacement was used to assess the robustness of edge weights, centrality stability was investigated through the case-dropping bootstrap using 2500 subsamples. To compare networks in different groups, the R package *NetworkComparisonTest* [73] was used. Based on 1000 data-driven permutations, network invariance (possible differences in edge weights) and global strength invariance (possible differences in the absolute sum of the network edge weights) were tested. In case of a significantly different network invariance, the Benjamini & Hochberg post-hoc test [74] was employed to investigate which edges were pairwise significantly different. All analyses were performed in the R software, version 4.3.0 (R Foundation for Statistical Computing, Vienna, Austria). The significance level was set to *p* < 0.05 for all statistical tests.

## Results

### Descriptive statistics

**Fig. 1:**
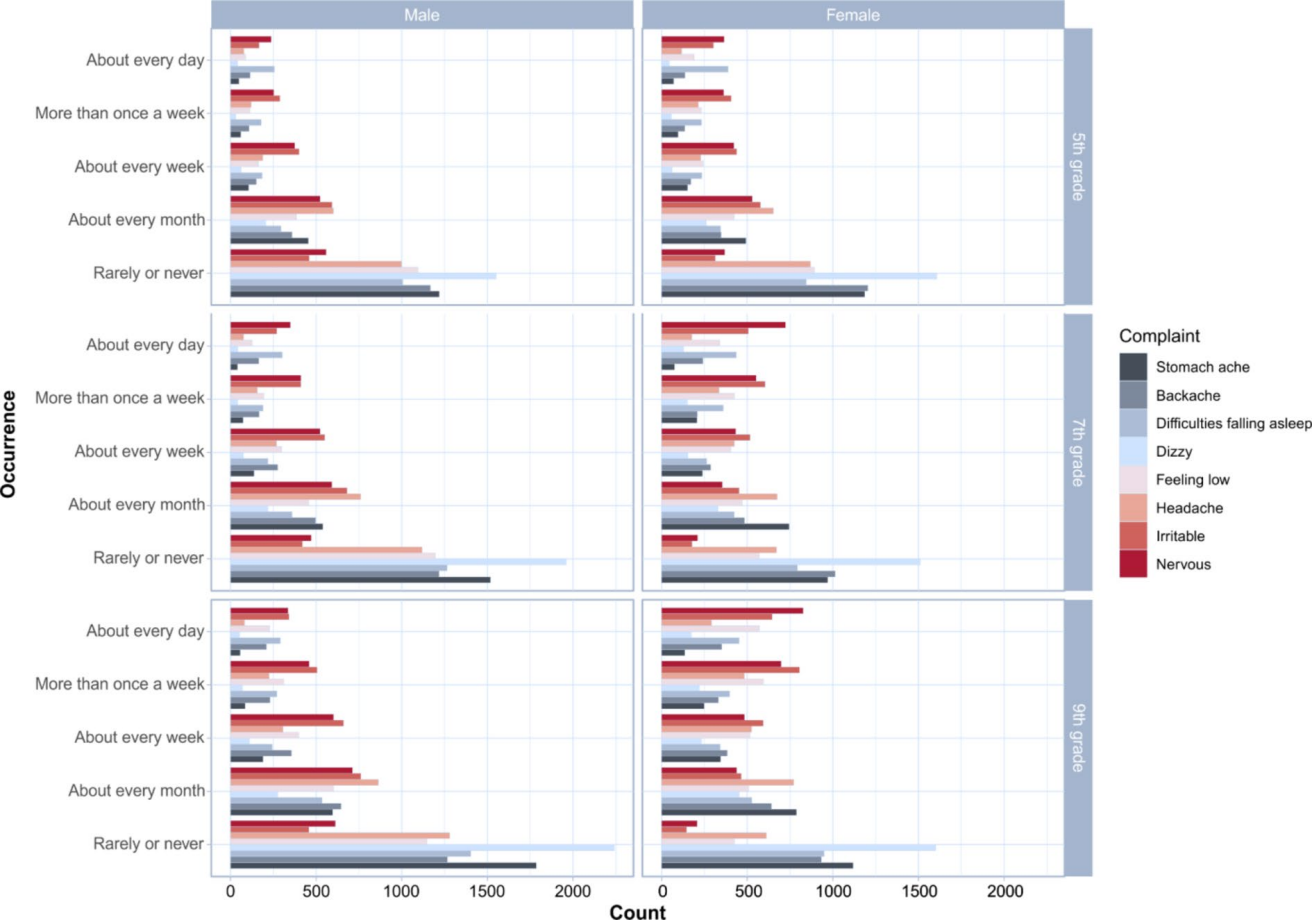
Prevalences of somatic and psychological complaints among Czech adolescents, stratified by sex and grade

Across all age groups, girls reported a higher prevalence of loneliness than boys (see Table 1). Overall, 25.9% of girls and 11.4% of boys reported feeling lonely most of the time or always. The youngest age group reported the lowest rates of loneliness (17.5% of girls and 7.9% of boys felt lonely most of the time or always), while the oldest age group reported the highest rates of loneliness (31.8% of girls and 15.5% of boys felt lonely most of the time or always). About a third of the boys (34.4%) and less than a quarter of the girls (22.7%) evaluated their overall health as excellent. The rate of reporting excellent health status changed with higher age, it increased in older boys and decreased in older girls (see Table 1). Psychological complaints were more common than somatic complaints in both gender groups. The most common psychological complaint in both genders was nervousness (49.2% of girls and 27.7% of boys felt nervous several times a week). The most prevalent somatic complaint reported by girls was headache (22.6% reported having headaches several times a week), while boys’ most common somatic complaint was backache (13.5% reported suffering from backache several times a week). In general, the prevalence of psychological complaints was higher in older age groups in both genders, while the prevalence of somatic complaints increased with age in girls and decreased with age or stagnated in boys. For more results, see Fig. 1 and Supplementary Table 1.

**Table 1:**
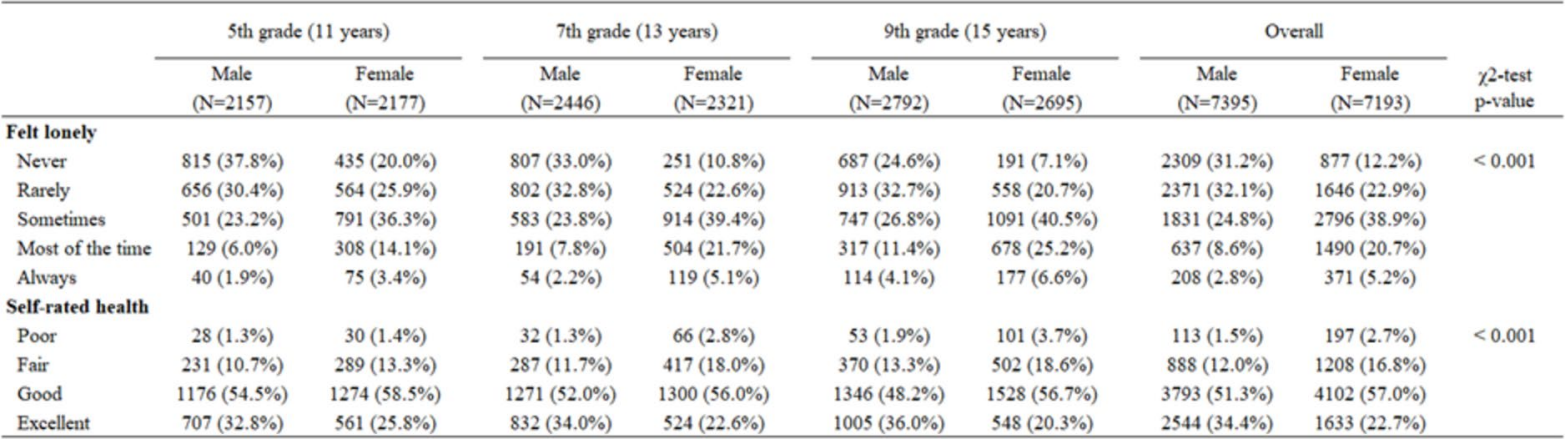
Descriptive characteristics of loneliness and overall health in Czech adolescents

### Network analysis

**Fig. 2:**
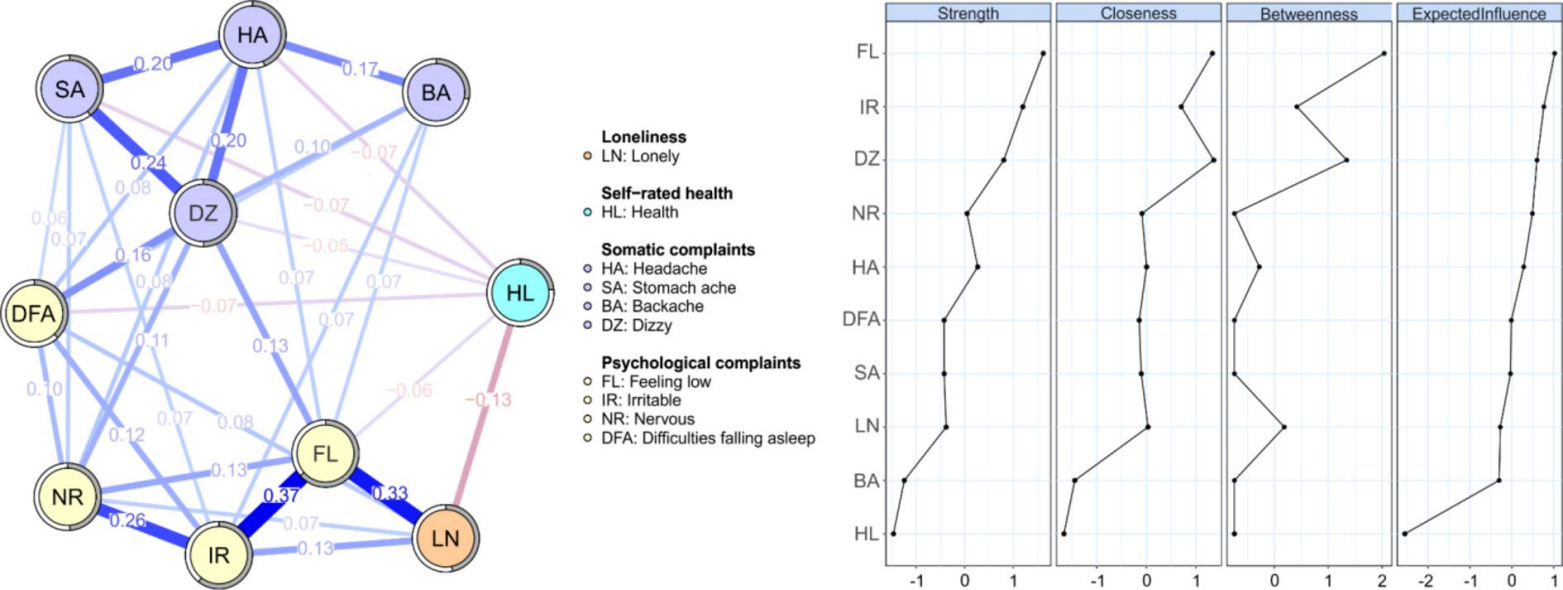
Overall network (left) and node centrality measures (right) were estimated on the full sample of Czech adolescents. For greater clarity, edge weights with absolute values below 0.05 were suppressed in the network plot. Positive edge weights are plotted in blue colour. Shaded areas (pies) surrounding nodes represent the predictability of the nodes. X-axes of centrality measures are scaled as Z-scores

As a first step, an overall network for the full sample was estimated (Fig. 2). The network revealed a negative direct relationship (i.e. non-zero-weight edge) between loneliness and health and positive direct relationships between loneliness and all of the individual psychological complaints. There were no strong direct links between loneliness and physical complaints. The edges stayed stable after bootstrapping. The original and bootstrapped edge weights are presented in Supplementary Fig. 1. The nodes with the highest centrality were feeling low, irritable and dizzy, the least central node was overall health. The stability of centrality measures after bootstrapping was sufficient (see Supplementary Fig. 1).

Next, based on the descriptive results, networks stratified by sex, FAS, and grade were estimated and compared. The Network Comparison Test did not confirm possible significant differences in the edge weights of networks stratified by sex or FAS (network invariance test statistic M = 0.07, *p* = 0.015 for sex; M = 0.10, *p* = 0.12 when comparing low and medium FAS, M = 0.06, *p* = 0.94 when comparing medium and high FAS). Therefore, only the results for networks stratified by grade are presented. Since sex and FAS may have an observed or unobserved influence on the relationships in the stratified networks, sex and FAS were included in the subsequent networks as control variables.

**Fig. 3:**
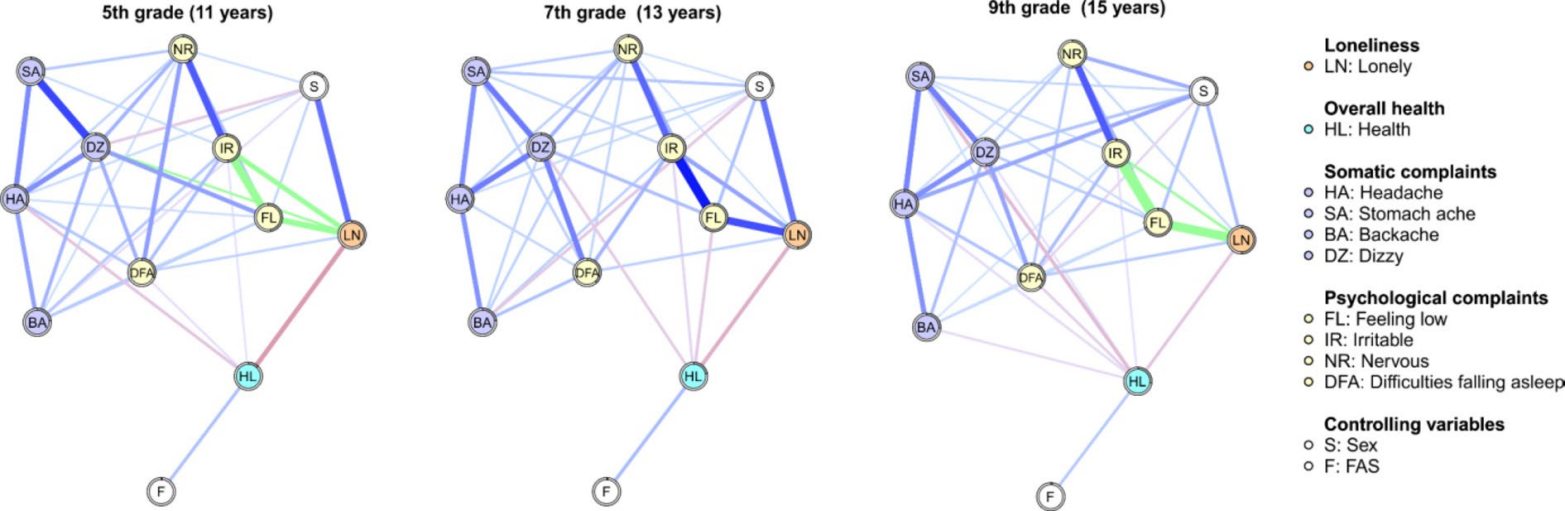
Networks stratified by grade of Czech adolescents, controlled for sex and FAS. For greater clarity, edge weights with absolute values below 0.05 were suppressed in the network plots. Positive edge weights are plotted in blue colour. Edges with significantly different weights between 5th and 9th grade are plotted in green. Thicker lines represent higher edge weights. To ensure comparability of the lines between the grades, the maximum line thickness in the plots was normed to the maximum absolute edge weight of the three networks. Shaded areas (pies) surrounding nodes represent the predictability of the nodes

**Fig. 4:**
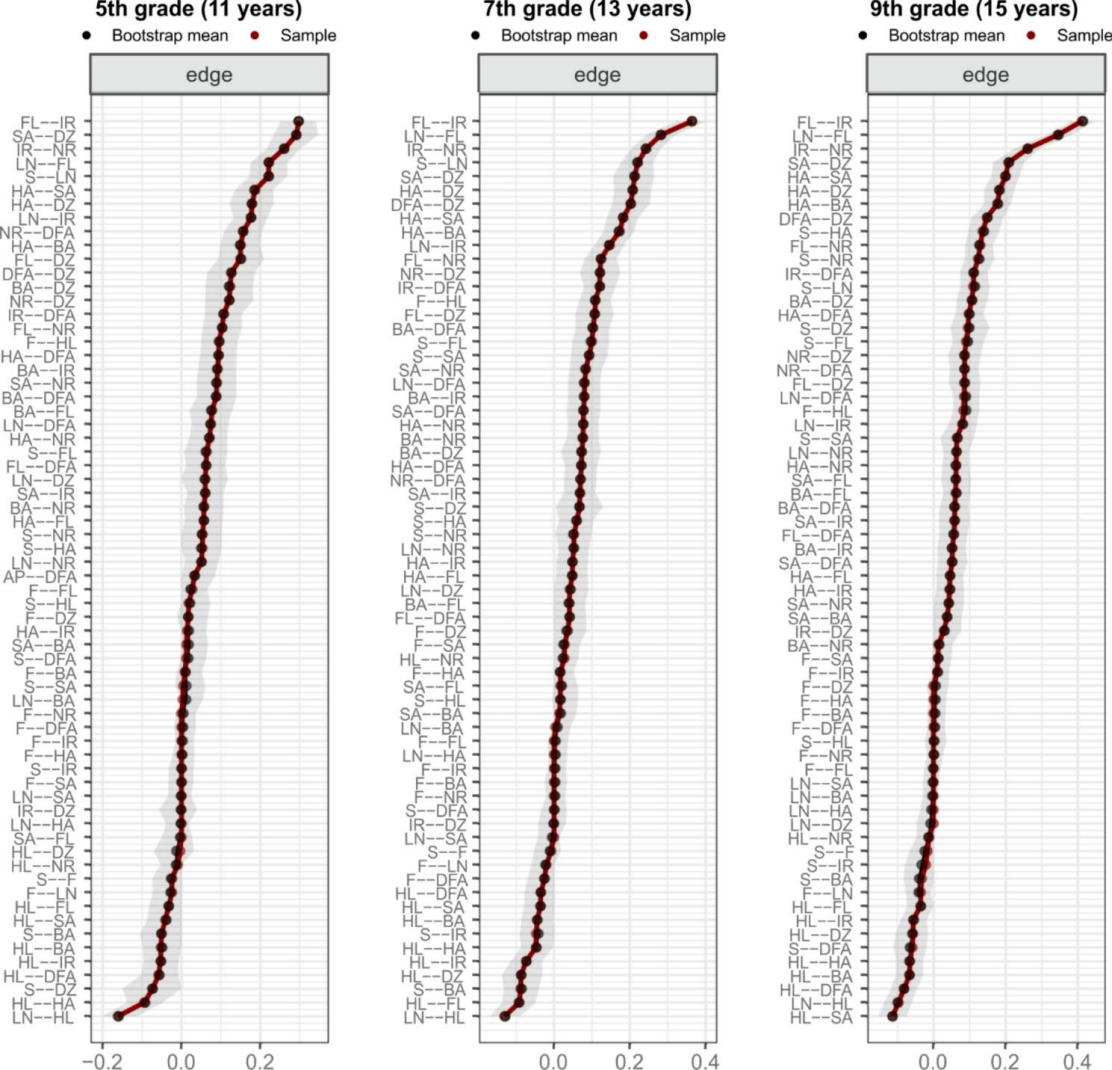
Bootstrapped edge weights for pairwise node comparisons in Czech adolescents stratified by grade. *LN* loneliness, *HL* health, *SA* stomach ache, *BA* backache, *DZ* dizzy, *FL* feeling low, *HA* headache, *IR* irritable, *NR* nervous, *DFA* difficulties falling asleep

The stratified networks (Fig. 3) unveiled that the strength of the negative direct relationship between loneliness and health in adolescents decreased with age. The strength of the positive link between loneliness and being irritable decreased as well. On the contrary, the positive link between loneliness and feeling low strengthened in higher age groups. In general, links between individual psychological complaints got stronger with higher age. There is an exception in the relationship between nervousness and difficulties falling asleep, where a moderately strong relationship in the youngest age group was almost diminished in higher age groups. The links between somatic complaints decreased their strength or stagnated with age. The edges of the stratified networks stayed stable after bootstrapping, see Fig. 4. The node with the highest centrality in all three grades was feeling low, the least central nodes were backache and overall health. The centrality measures after bootstrapping were considered sufficiently stable (see Supplementary Fig. 2).

### Network comparison test

The overall network invariance test showed that the edge weights significantly differed between all grades (5th versus 7th grade M = 0.14, *p* = 0.002; 7th versus 9th grade M = 0.11, *p* = 0.021; 5th versus 9th grade M = 0.17, *p* < 0.001). According to post hoc tests, there were no significantly different pairs of edges between 5th and 7th grade and 7th and 9th grade. When comparing 5th and 9th grades, 4 out of 45 pairs of edges (8.9%) significantly differed. These significantly different pairs were loneliness– feeling low (5th grade = 0.22, 9th grade = 0.35, *p* = 0.013), loneliness– irritability (5th grade = 0.18, 9th grade = 0.08, *p* = 0.013), loneliness– dizziness (5th grade = 0.06, 9th grade = 0, *p* = 0.028), and feeling low– irritability (5th grade = 0.30, 9th grade = 0.42, *p* = 0.013). The global strength invariance test did not reveal any significant differences in strength estimates between the grades (5th grade = 4.91, 7th grade = 5.22, 9th grade = 5.20, *p* > 0.05).

## Discussion

The objective of this study was to analyse the changes in loneliness among adolescents and examine how the associations between loneliness and mental and physical health vary across three age groups within the adolescent population. The results indicate that the strength of the association between loneliness and health decreases with age. Significant positive associations were found between loneliness, feeling low, and irritability, with no direct link between physical health complaints and loneliness but a possible indirect influence through mental health.

Research indicates that the relationship between loneliness and health outcomes varies across different age groups [13, 75–81]. Most longitudinal evidence shows that loneliness peaks in early adolescence and then decreases [13, 75, 79–81], although some subgroups demonstrate “high, reducing” or “low, increasing” patterns [76, 77]. One large-scale investigation, however, suggests that direct measures of loneliness may continue rising into the mid-20s, whereas social loneliness declines [78]. This observed decline in loneliness in later adolescence may also influence its relation to health outcomes [76, 77]. The decreasing strength of the relationship between loneliness and health in older adolescents may be attributed to the maturation of biological stress regulation mechanisms, such as improved hypothalamus-pituitary-adrenal (HPA) axis regulation. Previous research has demonstrated that dysregulation of the HPA axis is linked to loneliness [82–84]. Therefore, as adolescents mature, hormonal adjustments and brain development improve their ability to regulate and manage the stress associated with loneliness, reducing its negative relation with health [85, 86]. Another explanation for the decrease in the strength of the association is the improvement in social skills observed between younger and older adolescents. Research has shown that older children and adolescents generally have better social skills [87, 88], enabling them to maintain stronger social bonds with their peers and build new ones more easily [89, 90]. The stronger social bonds may reduce loneliness and decrease its negative association with health [91, 92]. Additionally, adopting and developing more effective coping mechanisms during adolescence can help regulate and significantly reduce the stress associated with loneliness and decrease its association with health [91, 93, 94].

Nevertheless, evidence of previous studies still remains mixed on the direct causality of loneliness on health or whether loneliness merely acts as a marker of underlying conditions [36, 37, 44, 46, 47, 49].Observational designs and confounding factors, such as socioeconomic status, existing health conditions, or the quality of social connections often obscure the direction of causality [45, 47, 48, 52]. While some propose biological pathways—such as stress and inflammation—to explain how loneliness negatively impacts health [50–52], others argue for further longitudinal research to clarify these uncertainties [33, 37, 49, 53].

The results showed a significantly different strength of associations between loneliness, feeling low and irritability, concerning the age of adolescents. Between the ages of 11 and 15 years, the link between loneliness and feeling low got significantly stronger, while the link between loneliness and irritability got weaker. At the same time, the link between feeling low and irritability increased its strength. Early adolescence is marked by emotional turbulence and sensitivity to social exclusion [95, 96], intensifying the need for social belonging. Feelings of loneliness can lead to significant emotional distress, including feeling low [97–99] or irritability [96, 100]. Adolescents’ need for social belonging can result in frustration and irritability when unfulfilled [96, 101, 102]. As adolescents mature, their emotional regulation and social coping mechanisms improve, reducing the impact of loneliness on their emotional state [91, 94]. Older adolescents develop better cognitive and emotional strategies to handle social stressors [93] and expand their social networks, which help mitigate loneliness and mental health impacts [91, 92, 103]. Cognitive maturity also allows adolescents to contextualise feelings of loneliness better, reducing the likelihood for those feelings to translate into irritability [56, 104].

Feeling low, characterised by depressive symptoms, is a common response symptom to loneliness, with studies showing that loneliness increases during adolescence and predicts depressive symptoms, with lasting negative effects into adulthood [35, 57, 105]. This is due to negative self-appraisals, increased stress reactivity leading to elevated cortisol levels, and social withdrawal reducing positive social reinforcement [1, 33, 98]. Additionally, research revealed that depressive symptoms increase during adolescence, with trajectories showing a rise to high levels in both boys and girls [106]. Low moods may distort cognitive processes, causing individuals to interpret social cues negatively and feel discouraged from social interaction, leading to increased loneliness [35, 107, 108]. Adolescents with depressive symptoms often struggle to engage in social activities [109, 110], leading to a reduction in social support networks and exacerbating feelings of loneliness [111, 112], creating a feedback loop that intensifies depressive symptoms.

Research found that irritability is a common symptom of pediatric depression, emphasising the strong link between irritability and depressive symptoms [113, 114]. Emotional dysregulation, which often accompanies feelings of low mood, increases sensitivity and frustration, leading to increased irritability [115, 116]. This dysregulation may cause individuals to react more intensely to stressors, making ordinary annoyances feel more severe [117, 118]. Additionally, studies have shown that irritable adolescents are more likely to experience major depressive episodes later in life, as chronic frustration and emotional volatility drain emotional resources and decrease the capacity for positive emotions [113, 119, 120]. Longitudinal research supports this notion, indicating that early irritability predicts later depressive symptoms [95, 121]. Consequently, as levels of depressive symptoms increase during this period [106], there is likely a corresponding increase in irritability among older adolescents.

The research findings also revealed an association between loneliness and difficulties falling asleep across all studied age groups. In concurrence with our findings, previous research also found that loneliness consistently predicts poorer sleep across different age groups [122–130]. Young students who feel generally less lonely benefit more from good sleep, experiencing lower next-day worry and stress, whereas loneliness disrupts this beneficial link [122]. Among older adults, emotional loneliness predicts worse sleep over time, partly via increased stress [123], and meta-analytic findings confirm reciprocal longitudinal associations between loneliness and sleep problems [124]. Both objective and subjective social isolation also correlate with poor sleep quality [125], with lonely individuals showing longer sleep latency, more nocturnal awakenings, and daytime fatigue [126, 127]. While some evidence suggests social isolation exerts a distinct or stronger influence on sleep than loneliness per se [128, 130], research on adolescents demonstrates loneliness can still disrupt sleep, particularly when mediated by problematic social network use and rumination [129].

However, although the analysis revealed no significant direct relationship between physical health complaints and loneliness, it indicated a possible indirect influence of physical health on loneliness via mental health issues namely feeling low and irritability. On the contrary to our findings, loneliness is consistently directly linked to higher rates of physical complaints, including headaches, stomach aches, and other psychosomatic symptoms [65, 131–136]. Children and adolescents who feel lonely often experience more frequent health issues such as backaches, headaches, stomach aches, and other psychosomatic symptoms, including increased medication use [65, 131, 135, 136]. Loneliness in combination with poor social support further impacts self-rated health and raises the likelihood of medication overuse [131, 132]. Individuals suffering from frequent migraines were found to be especially vulnerable, with emotional loneliness exacerbating headache impact during periods of social isolation [134]. Additionally, exposure to interpersonal violence further intensifies loneliness’s effect on recurrent headaches [133]. Physical health problems can exacerbate mental health conditions like depression and anxiety, contributing to loneliness [137–139]. Chronic physical issues create stress and psychological distress, fostering isolation and loneliness [140, 141]. Physical health limitations can reduce social interactions [105, 142], affecting mental well-being, and increasing irritability and low mood, both associated with loneliness [143, 144]. This disruption of social contact can have a negative influence on the ability to build and maintain strong social bonds and peer support structures, which may further exacerbate the impact on mental health leading to higher feelings of loneliness [145]. Altogether, these findings offer a comprehensive overview of changes in loneliness and its relationship to health among young adolescents.

Additionally, the network comparison test did not reveal any significant differences in sex. Meta-analytic evidence indicates that across the lifespan, mean levels of loneliness demonstrate comparable patterns between males and females, with effect sizes approaching zero, suggesting minimal gender-based variations in loneliness experiences [32, 146, 147]. This finding has been consistently replicated across diverse cultural contexts and age groups [148, 149]. However, significant differences emerge in self-disclosure patterns, with women demonstrating greater willingness to acknowledge and report feelings of loneliness compared to their male counterparts [150, 151]. This disparity may be attributed to sociocultural factors, including gender-specific socialization processes and differential stigma associated with expressing emotional vulnerability [32, 152]. The reluctance of men to report loneliness may be influenced by traditional masculinity norms and societal expectations regarding emotional expression [153, 154]. Overall, while there are minimal gender differences in loneliness across the lifespan [32, 146], specific contexts such as age, marital status, and cultural background reveal more pronounced differences [155, 156]. Women are generally more open to acknowledging loneliness [150, 151], and social networks play a crucial role in mitigating loneliness, especially for men [157, 158]. These differences in reporting feelings of loneliness have been documented in young adults and adolescents [159–162]. Research on the relationship between loneliness and gender in children and young adults reveals both similarities and differences across genders. While some studies suggest minimal differences [150, 163, 164], others highlight specific gender-related patterns [32, 158].

The COVID-19 pandemic marked a turning point in loneliness research. Although a meta-analysis by Ernst et al. [165] confirmed an overall increase in loneliness during this period, the surge was less dramatic than some media suggested. At the same time, Xiao and Dang (2023) emphasize the substantial heterogeneity in Ernst et al.’s findings [165] and advocate for considering regional contexts [166]. Indeed, several studies focusing on the pandemic have reported a significant increase in loneliness among emerging adults (18–25 years) [25, 167]. In the Czech Republic specifically, stringent social-distancing measures and repeated school closures greatly reduced adolescents’ opportunities for in-person interaction [165, 168]. This isolation was closely tied to mental health challenges such as depression, anxiety, and stress [18, 168–170]. These findings highlight the profound impact of increased loneliness during the COVID-19 pandemic on mental well-being of adolescents.

### Implications

#### Implications for research

The present study suggests several directions for future research. First, researchers should prioritise longitudinal studies to better understand how the relationship between loneliness and health evolves across different developmental stages. This approach will help identify critical periods when interventions are most needed. Additionally, these longitudinal studies could benefit from advanced analytical methods like network analysis, which effectively capture the complex, bidirectional dynamics between loneliness and health outcomes. Second, future research should utilise mixed-methods approaches that combine quantitative data with qualitative insights. This combination can offer a more comprehensive understanding of how loneliness impacts adolescents’ mental and physical health. Third, future research should also incorporate contextual factors, including family dynamics, peer relationships, and school environments. Forth, future studies also need to consider vulnerable minority groups, such as immigrants or LGBTQ + communities. Understanding how these variables interact with loneliness and health can enable the development of more holistic and effective intervention strategies to support adolescent well-being. Finally, future research should extend our findings by using more comprehensive psychometric tools to explore relationships between loneliness and mental as well as physical health. For instance, further research should focus on how different aspects of loneliness (e.g. emotional and social) are related to mental and physical health outcomes.

#### Implications for practice

The findings on the relationship between loneliness and adolescent health can help to shape future school social work strategies by guiding both school-based interventions and family-oriented support. First, social workers can collaborate with teachers and school administrators to identify at-risk children, ensuring early interventions that address unique needs. Second, fostering peer engagement through structured group activities, peer mentoring, and pupil-led teams can improve adolescents’ social skills and sense of belonging. In addition to benefiting mental and physical health, these programs can also help reduce stress levels, which is vital given the biological mechanisms associated with loneliness. Additionally, parents or caregivers should be included in interventions. Encouraging regular family discussions about mental health and providing psychoeducational resources help parents recognize and respond to signs of loneliness early. Family bonding activities—such as shared mealtimes, game nights, or outings—reinforce emotional connections at home. Adolescents themselves can be taught evidence-based coping strategies (e.g., relaxation techniques, mindfulness practices) and healthy communication skills to manage stress and build stronger relationships. School social workers can also involve local community organizations to create spaces where young people can socialize, collaborate, and practice new skills in safe, structured environments. Effective intervention strategies might include group therapy, drama therapy, or topical educational programs (e.g., digital well-being, social media literacy) that address potential loneliness triggers. Previous Czech initiatives have included notable programs such as the National Pedagogy Institute’s launch of the web portal dusevnizdravi.edu.cz, designed to provide comprehensive support to schools, teachers, and parents in addressing mental health challenges among students [171]. Additionally, the National Institute of Mental Health (NUDZ), in collaboration with UNICEF and WHO, has implemented programs aimed at promoting mental health among children and adolescents, with a special focus on reaching marginalized communities [172]. These programs not only address mental health issues but also foster inclusivity and accessibility in mental health care for young people.

### Limitations

Our study has several limitations: first, the study’s cross-sectional design limits the ability to infer causality between loneliness and health outcomes. Second, reliance on self-reported measures may introduce response biases, as participants might underreport or overreport their feelings of loneliness and health complaints due to, e.g., social desirability or recall bias. Third, the study’s focus on a single country, the Czech Republic, may limit the generalizability of the findings to other cultural contexts where social norms may differ. Fourth, although the network analysis can capture complex relationships, it can not handle non-linear associations that could be potentially expected in some variables in the present study. Moreover, given that network analysis is relatively novel within psychological research, there are inherent uncertainties - particularly concerning the reliability and stability of the estimated edges. To address these concerns, we employed state-of‐the‐art bootstrap-based methods to assess the stability and accuracy of our parameter estimates [61]. Furthermore, a systematic review of network studies [173] suggested that networks estimated with a relatively high sample size (i.e. exceeding 61 observations per potential edge) provide sufficient evidence for concluding the presence or absence of the respective edges. Our study meets this requirement. Fifth, this study did not consider vulnerable minority groups, such as immigrants or LGBTQ + communities, which may be affected by higher rates of loneliness. Finally, the study employed single-item measures to assess loneliness, mental, and physical health. While such measures are practical for large-scale surveys and demonstrate acceptable validity [174, 175] as well as test-retest reliability [68, 174, 176], they may not be able to distinguish between more nuanced aspects of a construct under investigation. For instance, a single-item loneliness scale is not able to distinguish between the emotional and social aspects of loneliness. For all of these reasons, our results should be interpreted with caution.

## Conclusion

This study aimed to examine the developmental progression of loneliness in adolescents and to explore how the relationships between loneliness and mental and physical health outcomes differ across three specific age groups within the adolescent population. The results indicate that the strength of the association between loneliness and health decreases with age. However, the edge difference test revealed that these differences were not statistically significant. The findings also showed significant positive associations between loneliness, feeling low, and irritability, with the association between loneliness and irritability weakening with age. Furthermore, the network analysis indicated no significant direct association between loneliness and physical health complaints but suggested a possible indirect influence of loneliness through mental health issues, specifically irritability and feeling low. Future research, ideally of a longitudinal nature, is needed to verify the changes in relationships between loneliness and health outcomes.

## Electronic supplementary material

Below is the link to the electronic supplementary material.

Supplementary Material 1

Supplementary Material 2

Supplementary Material 3

## Data Availability

Data, programming scripts, and additional resources linked to this research can be accessed through the Open Science Framework (OSF) portal using the specified digital object identifier (DOI) at: https://doi.org/10.17605/OSF.IO/E6NYM.
