# Supplementary material for: Network analysis of loneliness, mental, and physical health in Czech adolescents
Source: Child Adolesc Psychiatry Ment Health. 2025 Mar 28;19:34. doi: 10.1186/s13034-025-00884-7 (PMC11954233; doi:10.1186/s13034-025-00884-7)
Supplement: Supplementary file 1 — Supplementary Material 1 [file 13034_2025_884_MOESM1_ESM.docx]

**Supplementary table 1** Descriptive characteristics of somatic and psychological complaints among Czech adolescents, stratified by sex and grade

|  | 5th grade (11 years) | |  | 7th grade (13 years) | |  | 9th grade (15 years) | |  | Overall | |  |
| --- | --- | --- | --- | --- | --- | --- | --- | --- | --- | --- | --- | --- |
|  | Male | Female |  | Male | Female |  | Male | Female |  | Male | Female | χ2-test  *p*-value |
| **Somatic complaints** | (N=2157) | (N=2177) |  | (N=2446) | (N=2321) |  | (N=2792) | (N=2695) |  | (N=7395) | (N=7193) |  |
| **Headache** |  |  |  |  |  |  |  |  |  |  |  |  |
| Rarely or never | 999 (46.3%) | 869 (39.9%) |  | 1119 (45.7%) | 671 (28.9%) |  | 1280 (45.8%) | 612 (22.7%) |  | 3398 (46.0%) | 2152 (29.9%) | < 0.001 |
| About every month | 601 (27.9%) | 653 (30.0%) |  | 760 (31.1%) | 675 (29.1%) |  | 864 (30.9%) | 772 (28.6%) |  | 2225 (30.1%) | 2100 (29.2%) |  |
| About every week | 189 (8.8%) | 229 (10.5%) |  | 269 (11.0%) | 425 (18.3%) |  | 307 (11.0%) | 526 (19.5%) |  | 765 (10.3%) | 1180 (16.4%) |  |
| More than once a week | 121 (5.6%) | 215 (9.9%) |  | 157 (6.4%) | 336 (14.5%) |  | 226 (8.1%) | 483 (17.9%) |  | 504 (6.8%) | 1034 (14.4%) |  |
| About every day | 79 (3.7%) | 118 (5.4%) |  | 77 (3.1%) | 177 (7.6%) |  | 82 (2.9%) | 292 (10.8%) |  | 238 (3.2%) | 587 (8.2%) |  |
| **Stomach ache** |  |  |  |  |  |  |  |  |  |  |  |  |
| Rarely or never | 1219 (56.5%) | 1186 (54.5%) |  | 1518 (62.1%) | 970 (41.8%) |  | 1786 (64.0%) | 1119 (41.5%) |  | 4523 (61.2%) | 3275 (45.5%) | < 0.001 |
| About every month | 455 (21.1%) | 493 (22.6%) |  | 539 (22.0%) | 745 (32.1%) |  | 597 (21.4%) | 788 (29.2%) |  | 1591 (21.5%) | 2026 (28.2%) |  |
| About every week | 106 (4.9%) | 152 (7.0%) |  | 138 (5.6%) | 239 (10.3%) |  | 190 (6.8%) | 345 (12.8%) |  | 434 (5.9%) | 736 (10.2%) |  |
| More than once a week | 60 (2.8%) | 97 (4.5%) |  | 74 (3.0%) | 207 (8.9%) |  | 86 (3.1%) | 249 (9.2%) |  | 220 (3.0%) | 553 (7.7%) |  |
| About every day | 50 (2.3%) | 71 (3.3%) |  | 41 (1.7%) | 76 (3.3%) |  | 57 (2.0%) | 136 (5.0%) |  | 148 (2.0%) | 283 (3.9%) |  |
| **Backache** |  |  |  |  |  |  |  |  |  |  |  |  |
| Rarely or never | 1168 (54.1%) | 1205 (55.4%) |  | 1218 (49.8%) | 1014 (43.7%) |  | 1267 (45.4%) | 933 (34.6%) |  | 3653 (49.4%) | 3152 (43.8%) | < 0.001 |
| About every month | 360 (16.7%) | 348 (16.0%) |  | 497 (20.3%) | 484 (20.9%) |  | 646 (23.1%) | 642 (23.8%) |  | 1503 (20.3%) | 1474 (20.5%) |  |
| About every week | 151 (7.0%) | 172 (7.9%) |  | 276 (11.3%) | 287 (12.4%) |  | 356 (12.8%) | 384 (14.2%) |  | 783 (10.6%) | 843 (11.7%) |  |
| More than once a week | 109 (5.1%) | 136 (6.2%) |  | 168 (6.9%) | 209 (9.0%) |  | 231 (8.3%) | 332 (12.3%) |  | 508 (6.9%) | 677 (9.4%) |  |
| About every day | 115 (5.3%) | 137 (6.3%) |  | 165 (6.7%) | 242 (10.4%) |  | 210 (7.5%) | 352 (13.1%) |  | 490 (6.6%) | 731 (10.2%) |  |
| **Dizzy** |  |  |  |  |  |  |  |  |  |  |  |  |
| Rarely or never | 1551 (71.9%) | 1607 (73.8%) |  | 1961 (80.2%) | 1510 (65.1%) |  | 2241 (80.3%) | 1600 (59.4%) |  | 5753 (77.8%) | 4717 (65.6%) | < 0.001 |
| About every month | 206 (9.6%) | 261 (12.0%) |  | 219 (9.0%) | 329 (14.2%) |  | 275 (9.8%) | 452 (16.8%) |  | 700 (9.5%) | 1042 (14.5%) |  |
| About every week | 62 (2.9%) | 61 (2.8%) |  | 75 (3.1%) | 153 (6.6%) |  | 110 (3.9%) | 233 (8.6%) |  | 247 (3.3%) | 447 (6.2%) |  |
| More than once a week | 31 (1.4%) | 57 (2.6%) |  | 42 (1.7%) | 151 (6.5%) |  | 69 (2.5%) | 221 (8.2%) |  | 142 (1.9%) | 429 (6.0%) |  |
| About every day | 40 (1.9%) | 44 (2.0%) |  | 43 (1.8%) | 129 (5.6%) |  | 53 (1.9%) | 173 (6.4%) |  | 136 (1.8%) | 346 (4.8%) |  |

(*Supplementary* *table 1 continued*)

|  | 5th grade (11 years) | |  | 7th grade (13 years) | |  | 9th grade (15 years) | |  | Overall | |  |
| --- | --- | --- | --- | --- | --- | --- | --- | --- | --- | --- | --- | --- |
|  | Male | Female |  | Male | Female |  | Male | Female |  | Male | Female | χ2-test  *p*-value |
| **Psychological complaints** | (N=2157) | (N=2177) |  | (N=2446) | (N=2321) |  | (N=2792) | (N=2695) |  | (N=7395) | (N=7193) |  |
| **Feeling low** |  |  |  |  |  |  |  |  |  |  |  |  |
| Rarely or never | 1094 (50.7%) | 892 (41.0%) |  | 1196 (48.9%) | 570 (24.6%) |  | 1148 (41.1%) | 425 (15.8%) |  | 3438 (46.5%) | 1887 (26.2%) | < 0.001 |
| About every month | 385 (17.8%) | 424 (19.5%) |  | 457 (18.7%) | 471 (20.3%) |  | 602 (21.6%) | 509 (18.9%) |  | 1444 (19.5%) | 1404 (19.5%) |  |
| About every week | 163 (7.6%) | 244 (11.2%) |  | 298 (12.2%) | 405 (17.4%) |  | 398 (14.3%) | 517 (19.2%) |  | 859 (11.6%) | 1166 (16.2%) |  |
| More than once a week | 114 (5.3%) | 233 (10.7%) |  | 195 (8.0%) | 425 (18.3%) |  | 311 (11.1%) | 596 (22.1%) |  | 620 (8.4%) | 1254 (17.4%) |  |
| About every day | 88 (4.1%) | 190 (8.7%) |  | 127 (5.2%) | 340 (14.6%) |  | 227 (8.1%) | 571 (21.2%) |  | 442 (6.0%) | 1101 (15.3%) |  |
| **Irritable** |  |  |  |  |  |  |  |  |  |  |  |  |
| Rarely or never | 460 (21.3%) | 314 (14.4%) |  | 420 (17.2%) | 178 (7.7%) |  | 458 (16.4%) | 146 (5.4%) |  | 1338 (18.1%) | 638 (8.9%) | < 0.001 |
| About every month | 593 (27.5%) | 577 (26.5%) |  | 681 (27.8%) | 453 (19.5%) |  | 761 (27.3%) | 465 (17.3%) |  | 2035 (27.5%) | 1495 (20.8%) |  |
| About every week | 401 (18.6%) | 439 (20.2%) |  | 550 (22.5%) | 517 (22.3%) |  | 660 (23.6%) | 594 (22.0%) |  | 1611 (21.8%) | 1550 (21.5%) |  |
| More than once a week | 289 (13.4%) | 407 (18.7%) |  | 411 (16.8%) | 605 (26.1%) |  | 506 (18.1%) | 805 (29.9%) |  | 1206 (16.3%) | 1817 (25.3%) |  |
| About every day | 167 (7.7%) | 303 (13.9%) |  | 270 (11.0%) | 507 (21.8%) |  | 342 (12.2%) | 646 (24.0%) |  | 779 (10.5%) | 1456 (20.2%) |  |
| **Nervous** |  |  |  |  |  |  |  |  |  |  |  |  |
| Rarely or never | 559 (25.9%) | 369 (17.0%) |  | 472 (19.3%) | 211 (9.1%) |  | 613 (22.0%) | 208 (7.7%) |  | 1644 (22.2%) | 788 (11.0%) | < 0.001 |
| About every month | 524 (24.3%) | 530 (24.3%) |  | 592 (24.2%) | 355 (15.3%) |  | 713 (25.5%) | 439 (16.3%) |  | 1829 (24.7%) | 1324 (18.4%) |  |
| About every week | 375 (17.4%) | 423 (19.4%) |  | 524 (21.4%) | 433 (18.7%) |  | 602 (21.6%) | 484 (18.0%) |  | 1501 (20.3%) | 1340 (18.6%) |  |
| More than once a week | 253 (11.7%) | 363 (16.7%) |  | 411 (16.8%) | 553 (23.8%) |  | 460 (16.5%) | 699 (25.9%) |  | 1124 (15.2%) | 1615 (22.5%) |  |
| About every day | 238 (11.0%) | 366 (16.8%) |  | 350 (14.3%) | 724 (31.2%) |  | 336 (12.0%) | 827 (30.7%) |  | 924 (12.5%) | 1917 (26.7%) |  |
| **Difficulties falling asleep** |  |  |  |  |  |  |  |  |  |  |  |  |
| Rarely or never | 1006 (46.6%) | 845 (38.8%) |  | 1265 (51.7%) | 793 (34.2%) |  | 1403 (50.3%) | 949 (35.2%) |  | 3674 (49.7%) | 2587 (36.0%) | < 0.001 |
| About every month | 295 (13.7%) | 345 (15.8%) |  | 360 (14.7%) | 424 (18.3%) |  | 535 (19.2%) | 527 (19.6%) |  | 1190 (16.1%) | 1296 (18.0%) |  |
| About every week | 185 (8.6%) | 235 (10.8%) |  | 219 (9.0%) | 264 (11.4%) |  | 244 (8.7%) | 342 (12.7%) |  | 648 (8.8%) | 841 (11.7%) |  |
| More than once a week | 179 (8.3%) | 233 (10.7%) |  | 190 (7.8%) | 360 (15.5%) |  | 271 (9.7%) | 398 (14.8%) |  | 640 (8.7%) | 991 (13.8%) |  |
| About every day | 256 (11.9%) | 388 (17.8%) |  | 303 (12.4%) | 436 (18.8%) |  | 291 (10.4%) | 453 (16.8%) |  | 850 (11.5%) | 1277 (17.8%) |  |
