# Supplementary material for: Network analysis of loneliness, mental, and physical health in Czech adolescents
Source: Child Adolesc Psychiatry Ment Health. 2025 Mar 28;19:34. doi: 10.1186/s13034-025-00884-7 (PMC11954233; doi:10.1186/s13034-025-00884-7)
Supplement: Supplementary file 2 — Supplementary Material 2 [file 13034_2025_884_MOESM2_ESM.docx]

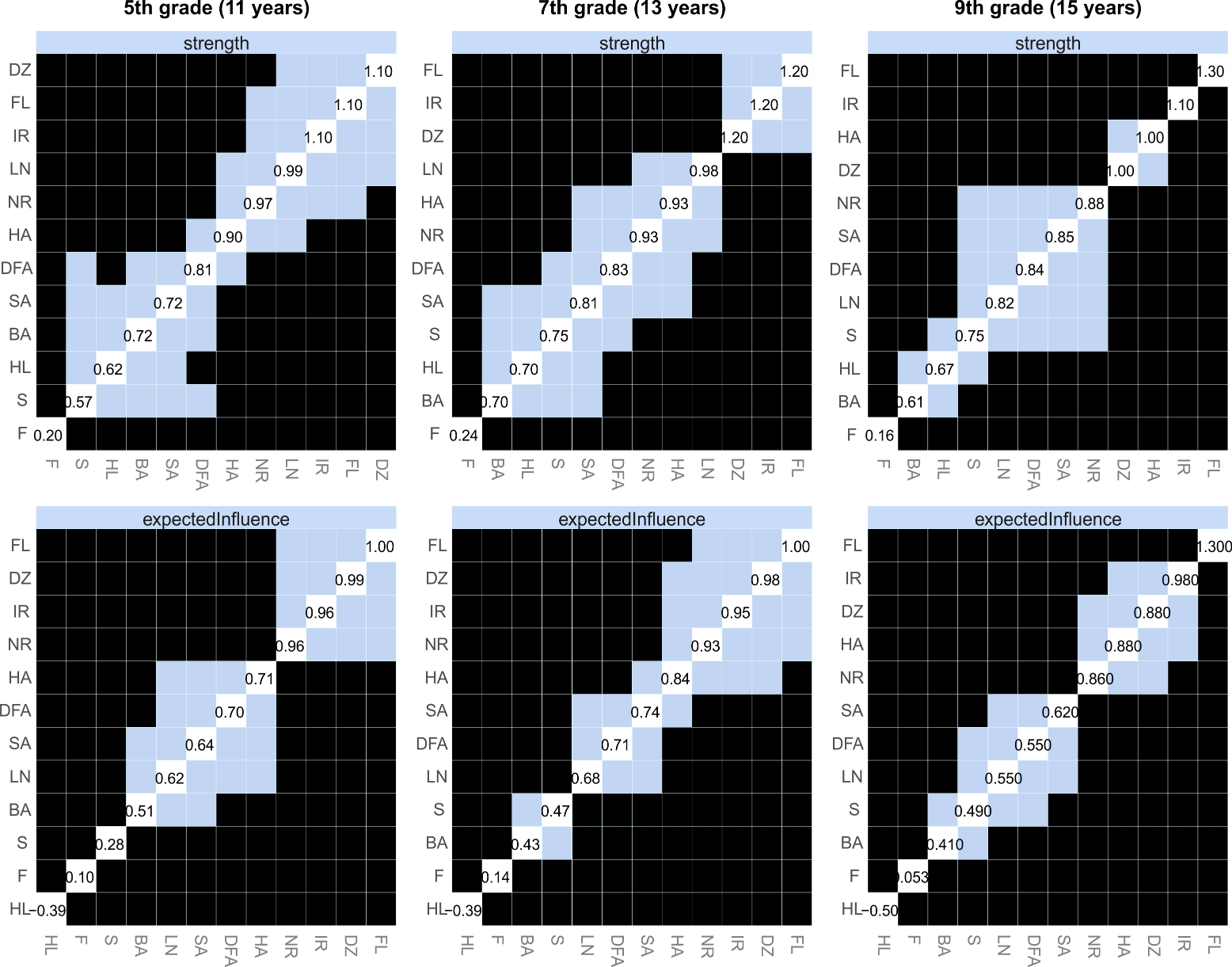


**Supplementary fig. 2** Bootstrapped stability of the centrality measures Strength and Expected Influence in Czech adolescents stratified by grade. Abbreviations: LN = loneliness, HL = health, SA = stomach ache, BA = backache, DZ = dizzy, FL = feeling low, HA = headache, IR = irritable, NR = nervous, DFA = difficulties falling asleep
