# Supplementary material for: Network analysis of loneliness, mental, and physical health in Czech adolescents
Source: Child Adolesc Psychiatry Ment Health. 2025 Mar 28;19:34. doi: 10.1186/s13034-025-00884-7 (PMC11954233; doi:10.1186/s13034-025-00884-7)
Supplement: Supplementary file 3 — Supplementary Material 3 [file 13034_2025_884_MOESM3_ESM.docx]

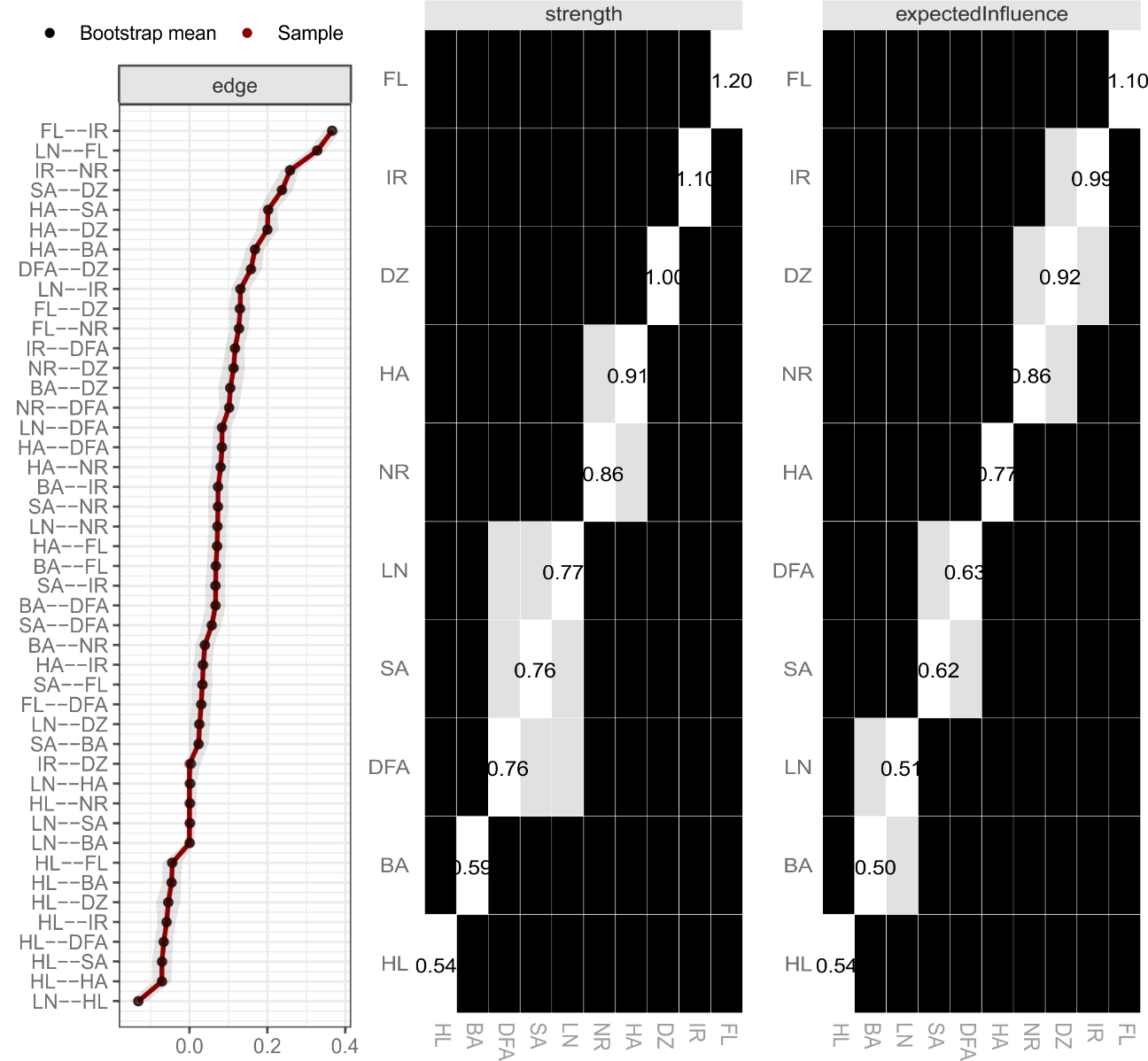


**Supplementary fig. 1** Bootstrapped stability of edge weights (left) and centrality measures (right) estimated on the full sample of Czech adolescents. Abbreviations: LN = loneliness, HL = health, SA = stomach ache, BA = backache, DZ = dizzy, FL = feeling low, HA = headache, IR = irritable, NR = nervous, DFA = difficulties falling asleep
